# Motivational interviewing for screening and feedback and encouraging lifestyle changes to reduce relative weight in 4-8 year old children: design of the MInT study

**DOI:** 10.1186/1471-2458-10-271

**Published:** 2010-05-24

**Authors:** Rachael W Taylor, Deirdre Brown, Anna M Dawson, Jill Haszard, Adell Cox, Elaine A Rose, Barry J Taylor, Kim Meredith-Jones, Lee Treacy, Jim Ross, Sheila M William

**Affiliations:** 1 Department of Medical and Surgical Sciences, University of Otago, Dunedin, New Zealand; 2 School of Psychology, Victoria University of Wellington, Wellington, New Zealand; 3 Department of Women's and Children's Health, University of Otago, Dunedin, NZ; 4 Department of Human Nutrition, University of Otago, Dunedin, NZ; 5 Pediatrics Services, Otago District Health Board, Dunedin, NZ; 6 School of Physical Education, University of Otago, Dunedin, NZ; 7 Department of General Practice, University of Otago, Dunedin, NZ; 8 Department of Preventive and Social Medicine, University of Otago, Dunedin, NZ

## Abstract

**Background:**

Because parental recognition of overweight in young children is poor, we need to determine how best to inform parents that their child is overweight in a way that enhances their acceptance and supports motivation for positive change. This study will assess 1) whether weight feedback delivered using motivational interviewing increases parental acceptance of their child's weight status and enhances motivation for behaviour change, and 2) whether a family-based individualised lifestyle intervention, delivered primarily by a MInT mentor with limited support from "expert" consultants in psychology, nutrition and physical activity, can improve weight outcomes after 12 and 24 months in young overweight children, compared with usual care.

**Methods/Design:**

1500 children aged 4-8 years will be screened for overweight (height, weight, waist, blood pressure, body composition). Parents will complete questionnaires on feeding practices, physical activity, diet, parenting, motivation for healthy lifestyles, and demographics. Parents of children classified as overweight (BMI ≥ CDC 85^th^) will receive feedback about the results using Motivational interviewing or Usual care. Parental responses to feedback will be assessed two weeks later and participants will be invited into the intervention. Additional baseline measurements (accelerometry, diet, quality of life, child behaviour) will be collected and families will be randomised to Tailored package or Usual care. Parents in the Usual care condition will meet once with an advisor who will offer general advice regarding healthy eating and activity. Parents in the Tailored package condition will attend a single session with an "expert team" (MInT mentor, dietitian, physical activity advisor, clinical psychologist) to identify current challenges for the family, develop tailored goals for change, and plan behavioural strategies that best suit each family. The mentor will continue to provide support to the family via telephone and in-person consultations, decreasing in frequency over the two-year intervention. Outcome measures will be obtained at baseline, 12 and 24 months.

**Discussion:**

This trial offers a unique opportunity to identify effective ways of providing feedback to parents about their child's weight status and to assess the efficacy of a supportive, individualised early intervention to improve weight outcomes in young children.

**Trial registration:**

Australian New Zealand Clinical Trials Registry ACTRN12609000749202

## Background

There is no doubt that overweight and obesity remain a significant problem for New Zealand children. Almost one in three New Zealand youngsters are affected by excess body weight, and this prevalence has remained stable over the past five years [1]. The optimal age to intervene and reduce excess weight has yet to be established, but there is currently considerable interest in targeting younger children [2], based on the premise that behavioural patterns that may be encouraging weight gain might be more easily altered at this age rather than becoming firmly entrenched.

Intervening in young children is complicated by the fact that parents of younger children, particularly preschoolers, have considerable difficulty recognising their children as overweight, unless it is particularly severe [3-8]. Moreover, few parents of young overweight children express concern about their child's weight [3,5,7] and are more likely to be concerned that their child is underweight, despite very few children being so [4,7]. Mothers of young overweight children only perceived weight to be an issue if their child was being teased or was limited in their physical capacity for exercise [4]. Thus parents of young overweight children are unlikely to seek help if the issue is not recognised [9]. The advent of screening initiatives provides the opportunity to proactively discuss with parents the weight status of their child. If screening is to occur however, ethical considerations dictate that participants must be informed of the results and that effective pathways for supporting parents to introduce positive changes are available [10]. From 2008, the height and weight of every 4-year-old child in New Zealand will be ascertained prior to them starting school as part of the new B4 School check [11]. Perhaps because weight status is an emotive issue, it has been suggested that parents only receive information regarding their child's height and weight, rather than be informed that their child is actually overweight [11].

This approach misses a prime opportunity to intervene in a positive manner at an age where behaviour change may be more achievable. Before this can occur however, we need to find the best way of informing parents of young children that their child is overweight so that 1) they are able to accept and understand the information and the associated risks of their child's weight status, and 2) their motivation and ability to make the behavioural changes required is maximised [12]. Despite increasing use of routine screening for overweight worldwide, little research has examined how parents react to receiving this information. Parents of overweight preschool-aged children who received information regarding their child's weight status in the form of a report card detailing whether their child was normal weight, at risk of overweight or overweight [13] were far more likely (P < 0.001) to be upset about the feedback than parents of overweight children who received a report card detailing height and weight only [14]. Furthermore, half of the parents in the former group rejected the classification of their child as being overweight, saying it did not change their perception of the child's weight and only 24% of parents with overweight children and 6% of those with children classified as at risk of overweight felt that action regarding their child's weight was required [14]. In contrast, a Canadian study in preschoolers [15] showed that 85% of parents of overweight children were happy with the information provided during the measurement process, although it was unclear what feedback was given apart from plotting the child's weight-for-height. Work in school-aged children demonstrates that the majority of parents want to know the weight status of their child [16,17] even though large proportions of children (both normal weight and overweight) may feel uncomfortable discussing the results with their parents [16].

Studies also highlight a common reluctance by medical professionals to broach the subject of overweight with patients and families and concern that raising this issue would negatively impact the relationship with their patients [18,19]. Focus groups undertaken with general practitioners and practice nurses as background work for this project showed that they felt very worried about the impact that this might have on the parents (increasing parental feelings of blame and guilt; concern about parents initiating diets or food restriction; and concern that parents may change doctors) and on the child (starting to diet, self-esteem and pressure to lose weight). Some health professionals showed concern related to using labels and terms to describe weight, and in particular, the term obese was described unfavourably. Work from focus groups with parents showed concern about how weight information might be discussed with them and stressed how important it was not to feel judged or blamed by the person giving the information. Parents did want to be given weight information and to be told if a weight problem existed, but some raised strong concerns about the terms overweight and obese (Dawson, unpublished). Interestingly, another recent study reported that parents of young children preferred the terms overweight and obese, as long as sufficient rationale was provided for using them [20].

One way of reducing the negative consequences that might result from discussing weight status is to use Motivational Interviewing (MI) during consultations. MI is a non-judgemental, guided, empathetic style of counselling, first developed as a brief intervention for problem drinking and other addictive behaviours [21]. MI has been the focus of ongoing development, research and application to a wide range of contexts where behaviour change (reduction of problem behaviours, increase of health promotion behaviours, or maintenance of health behaviours) is of interest [22-24]. The creators of MI describe it as "a client-centered, directive method for enhancing intrinsic motivation to change by exploring and resolving ambivalence" [21]. MI has been largely developed from clinical practice as a brief intervention and more recently, self-determination theory (SDT) [25] has been proposed to conceptualise and account for how MI works [26]. SDT aims to explain the causes, processes and outcomes of human motivation. SDT describes a continuum of motivation that reflects whether the regulation of behaviour change is more or less self-determined. Non self-determined motivation (also termed controlled behavioural regulation) reflects that the individual feels pressured or controlled to behave in a particular manner as a result of external pressures (e.g. to obtain rewards, avoid punishment, or satisfy someone else such as a health professional) or internal pressures (a sense of obligation, to avoid a sense of guilt). Self-determined motivation (also termed autonomous behavioural regulation) reflects that the decision to act originates from within the individual. This is as a result of the personal importance attached to the outcomes of the behaviour, an acknowledgement of the inherent value and enjoyment that the behaviour provides and that engaging in the behaviour is consistent with personal beliefs and sense of self [27].

SDT states [25] and literature supports [28,29] that self-determined or autonomously motivated behaviours are more likely to result in long-lasting change, rather than non self-determined or behaviours controlled by external forces which will only last as long as the pressure is in place [30]. Consequently, it is important to foster more self-determined motivation to encourage successful behaviour change. According to SDT, there are 3 main psychological needs that influence motivation [26]; these are autonomy (the need to feel like you have a choice, volition, and an internal perceived locus of causality), competence (the need to feel you have the ability to make the change) and relatedness (the need to feel connected to others). The extent to which these needs are supported will determine the level of self-determined motivation.

It has been suggested that the four general principles of MI foster our psychological needs and therefore promote more autonomous or self-determined motivation for change [26]. By *expressing empathy*, practitioners adopt a client-centred approach aimed at understanding a client's experience and developing a collaborative relationship. This contributes to a sense of relatedness in the client. By *supporting self-efficacy *through emphasising the client's choice and responsibility for change and highlighting skills, resources and previous successful experiences, and by *rolling with resistance *through encouraging the client to develop their own responses or solutions to perceived obstacles, both autonomy and competence are emphasised. Finally, by *developing discrepancy*, through helping the client to explore differences between their current situation and their expectations or ideals for the future, autonomy and an internal locus of causality is encouraged. Others [31] have shown that significant numbers of parents of overweight children may show no or limited interest in changing family behaviours to reduce child overweight. Thus, identifying such parents and utilising MI to generate and resolve their ambivalence towards making behaviour changes and supporting the creation and maintenance of self-determined motivation to initiate change is of immense interest.

While some success in treating childhood obesity has been observed, recent reviews highlight that much is still unknown regarding the most effective and sustainable options [2,32-34]. However, the need for a multidisciplinary approach seems mandatory, as does the need to engage the whole family, not just the overweight child [12,35-42]. Work in adults suggests that frequent contact may be a particularly important driver of successful weight management [43,44]. Considerable interest has also centred around the behaviour-modification strategies that best promote weight control [45]; goal-setting, positive reinforcement, problem solving, preventing relapse, self-monitoring, rewards, and identifying barriers are critical components of many weight interventions [39,42,46,47].

While many of these behaviour-modification strategies are also inherent within MI and an autonomy supportive framework aligned with SDT, the use of MI or SDT may be particularly intriguing for long-term weight management if practitioners are able to truly promote increased self-determined motivation [28,32]. While MI was developed to be a brief rather than sustained intervention, it may be particularly useful as a component of obesity treatment programmes. MI may increase engagement through enhancing motivation (especially self-determined) by exploring family perceptions of the child's weight, potential obstacles to change and sources of efficacy, and addressing ambivalence that may lead to families taking the default position of maintaining current behaviours (i.e. resistance) rather than engaging with change. Feelings of competence may be supported by exploring the client's own ideas and resources, and a sense of relatedness developed through a collaborative relationship with the practitioner that is client-centred and avoids the role of the expert telling the client what they need to do. Once engaged in the intervention, strategic use of MI with families at key stages (e.g., when progress slows or it is time to introduce a new behaviour goal) may assist in maintaining motivation (i.e. retention and completion). Indeed, several recent small studies have examined the efficacy of MI for effective weight management in children [32,48-50]. While these studies offer promise, all have been pilot initiatives and therefore have not been powered to detect clinically important changes in BMI.

A further advantage of MI over other techniques could be the ability to motivate parents who previously had not expressed any desire to invoke behaviour change with their families. The nature of recruitment in most obesity treatment studies means that only those parents who are highly motivated to seek treatment for their overweight children are likely to participate, and these children are generally very overweight. While the health implications of excess weight are most severe for those who are obese [51], in some ways the more mildly overweight group poses the most concern for future health care [52], given the far greater numbers of affected children and the high likelihood of progression to an obese state [53,54]. However, parents of mildly overweight young children are unlikely to seek treatment, given the general lack of awareness of overweight for this age group [3-8]. Thus typical methods of recruitment probably exclude large numbers of children who may benefit from appropriately designed interventions. Recruiting children via health screening provides an opportunity to access children at an earlier stage of unhealthy weight development, and parents with a greater range of motivation, for which MI offers considerable promise.

Thus the aim of our study is to develop a family-based approach derived from the SDT model of motivation and behaviour change utilising motivational interviewing for both feedback of screening results and to promote retention and continued involvement throughout the intervention, that is suitable for incorporation into the primary health care environment. Specifically, the study is aimed at reducing excessive weight gain in young children identified as overweight through health screening.

## Methods/Design

Our study has been designed to meet the needs of a screening programme for overweight in children, principally notification of outcomes and provision of suitable treatment pathways. Firstly, we wish to screen young children for overweight to compare the acceptability of information regarding children's weight status when delivered using motivational interviewing compared with receiving this information in a more general manner. Qualitative interviews with parents will explore their responses to this feedback and whether it produced any perceived harmful effects. Motivation to change behaviour as a result of the feedback will be assessed quantitatively by how many agree to participate in the intervention and from changes in their responses to the motivation questionnaires. Phase two aims to test the effectiveness of a programme that has been designed to be incorporated into primary care, has limited "expert" involvement, and works with each family individually to determine achievable long-term behavioural goals. The study has been approved by the Lower South Regional Ethics Committee (LRS/09/09/039).

### Phase 1: Screening and feedback

#### Subjects

**Figure 1:**
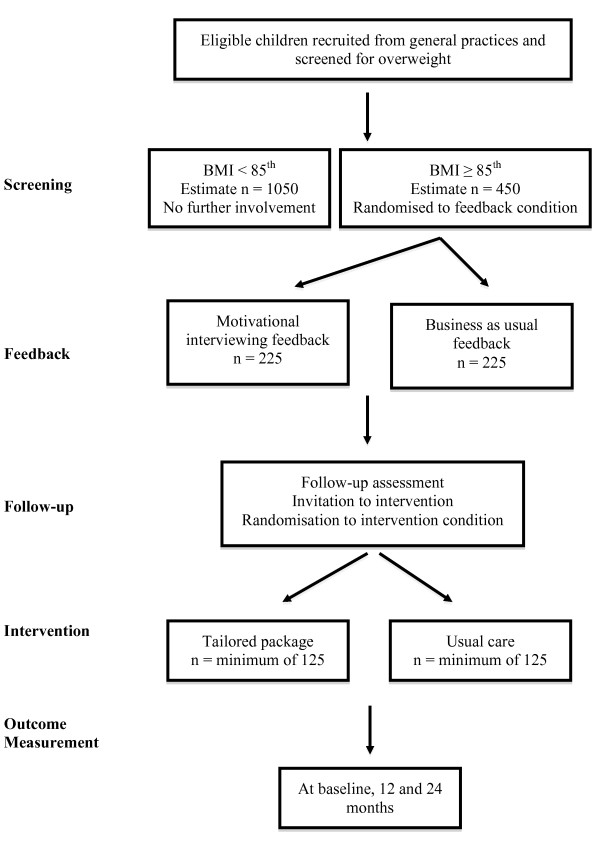
**Flow of participants through the study**.

All children aged 4-8 years enrolled at several Dunedin general practices will be invited to participate in a general health check. These practices represent a cross-section of the Dunedin population, both geographically and socio-economically and screening will continue until approximately 1500 children have been recruited. The flow of children through the study is shown in Figure 1.

#### Procedure

Height will be measured to the nearest 0.1 cm using a portable stadiometer, weight to the nearest 0.1 kg by Tanita electronic scales [55], and waist circumference (at the umbilicus) to the nearest 0.5 cm using a non-elastic tape. Body composition will be measured using bioelectrical impedance (BIA, Tanita BC-418) in all subjects [56,57] and in a random subsample by dual-energy x-ray absorptiometry (DXA, Lunar Prodigy) [58]. The DXA information will be used to validate the BIA results, particularly in the younger children given that the minimum age for the BC-418 is 7 years. Blood pressure will be measured by an automated sphygmomanometer (Dinamap: GE Medical Systems, Waukesha, WI) with children in a sitting position after a 5 minute rest [59]. All measures (except DXA) will be obtained in duplicate.

#### Health check questionnaire

Parents will complete the health check questionnaire which assesses parenting style and warmth/hostility, feeding practices, motivation for healthy lifestyles, dietary intake, physical activity and inactivity, parental weight status and demographics as described below. Parenting style will be assessed using the Parenting Scale [60,61] and warmth/hostility will be assessed using a 9-item questionnaire used in the Welsh Transition Project (Harold G, personal communication). The Comprehensive Feeding Practices questionnaire [62] has 12 subscales including monitoring, environment, use of food as a reward, restriction for weight and restriction for health. Motivation will be assessed using a modified version of Miller and Johnson's [63] motivational screening measure (MSM) to provide a brief assessment of three constructs of client's self-reported motivation for change; importance (It is important for me to...), ability (I could...) and commitment (I am trying to...). Measures of autonomous and controlled motivation will be assessed using the Treatment Self-Regulation questionnaire (TSRQ) [64,65]. Parents will report their child's typical level of physical activity and inactivity, intake of fruit/vegetables/sweetened drinks, and sleep habits. This information will be compared to current guidelines [66], and in relation to perceived risk (MSM above) and perceived need to change specific behaviours. Parents will also be asked to rate their child's weight (five possible options from very underweight to very overweight), be weighed themselves, and provide demographic information (ethnicity, socio-economic status).

#### Health check report card

The BMI percentile of each child will be calculated according to CDC reference norms [67] as per current paediatric guidelines [12,66]. Waist (cm) to height (cm) ratio (WHtR) will be calculated and compared with recommended levels [68]. All parents will receive a report card containing the results of the screening tests; height, weight, BMI, WHtR and blood pressure displayed graphically relative to age and sex, as well as reported intake of sweetened drinks and fruit/vegetables, daily hours of inactivity (screen time) and physical activity, and sleep habits, along with recommended guidelines for these behaviours. Children with BMI values < 85^th ^percentile will be identified as normal weight and will not participate further in the study. Children with BMI values ≥ 85^th ^percentile will be randomised to one of two feedback conditions; Usual care or Motivational interviewing (MI). Both feedback conditions will utilise the report cards as above and will be discussed with each family in face-to-face interviews. For those randomised to the Usual care feedback condition, the health advisor will discuss the results briefly with the families. In the motivational interviewing condition, the same report card will be used but the MInT mentor will use MI to discuss the findings in a way that allows parents to explore their reactions to the information and what they might be able to do in response. The feedback will utilise the Elicit-Provide-Elicit model of MI, first asking parents for their perception or current knowledge of their child's development in each area, providing feedback for the specific assessment measures taken, and then eliciting and exploring their reaction to this information using MI techniques and style. The interaction will focus in particular on eliciting any resistance or distress experienced by the parent with the goal of helping them to work through this and identify sources of motivation for change and the resources (internal and external) that they can access to be successful.

#### Follow-up interviews

Follow-up interviews will be held with parents two weeks after the feedback to assess whether any changes in motivation or behaviour had occurred, how the parents found the feedback and any issues arising from this, and whether any perceived benefit or harm arose from feedback. Several items from the Health check questionnaire will be readministered including the MSM, TSRQ, warmth/hostility questionnaire, brief behaviour questions, and selected subscales of the Comprehensive Feeding Practices Questionnaire. Additional questions address the parent's response to feedback as well as the child's response (if discussed with the parent). The parents will also complete the short-form Health Care Climate Questionnaire, which assesses the degree to which they felt the feedback was provided in an autonomy-supportive versus controlling manner [28].

### Phase 2: Treatment

When the follow-up feedback interview is complete, parents will be invited into the treatment phase. Those consenting to participate will be given additional baseline questionnaires as described below. Participants will then be randomised (stratified by feedback condition) to one of two intervention conditions; Usual care versus Tailored package.

#### Usual Care group

The family will meet once with the health advisor (30-60 minutes) who will offer general advice regarding healthy eating, physical activity, and parenting using existing public resources. If families in this group want any additional contact, they will be referred back to their general practice as per the "usual" care in that practice. Given the variability in "usual" care for childhood overweight, information will be sought to gauge if any additional contact occurred, how often, and who with (practice nurse versus GP).

#### Tailored Package group -- treatment phase

The *Tailored package *is modelled in part on our successful HEAT study [43] and from the literature [2,39,66,69] and is designed to be suitable for incorporation into primary care. Three main areas of interest will be assessed and targeted; dietary intake, physical activity/inactivity, and parenting/behaviour (Table 1). In the *Tailored Package *condition, parents will attend one session with a multi-disciplinary team (consultant session) then all further contact will be with their MInT mentor.

**Table 1:** Goals and target behaviours of interest.

Diet	Behaviour management
Making water the main drink	Stress management for parents
Eating more fruit and vegetables	Using attention and effective commands
Changing fast food choices	Using ground rules and rewards
Healthy snacks	Discipline and consequences
Appropriate portion size	Developing action plans
Family meals	

**Physical activity/inactivity**	**Other**
Motivating kids to be active	Helping children sleep
Reducing screen time	
Increasing moderate/vigorous activity	
Increasing family activity	

#### Consultant session

Information obtained from the screening, follow-up and baseline assessments (family structure, economic situation, dietary intake, physical activity, child behaviour, motivation, parental weight, parenting) will be used by the clinical psychologist to develop a formulation that is specific for each family. This formulation will provide an explanation of factors that may have contributed to the development of the child's weight, and may be maintaining the situation, as well as identifying strengths and resources in the family. The family will then meet with the "expert" team, consisting of the clinical psychologist, a dietitian, an exercise specialist and the MInT mentor to discuss and modify the formulation as appropriate and to reflect on the implications of this for possible goals for change. The main objective of this session is to assist the family in developing an understanding of their current situation, and to collaboratively identify areas in which they may wish to make changes in. Once the goals have been identified, the session will focus on developing an individualised plan for each family consisting of strategies that they can use to achieve the goals they have identified.

#### Mentor sessions - timing

The MInT mentor will then become the main contact for each family. To aid in establishing new routines during the first phase of the treatment period (4 months) the mentor will contact the family each week, using an alternating but flexible schedule of in-person consultations and telephone calls. Frequency of contact will be gradually reduced over the subsequent 20 months of the intervention (fortnightly for months 5-8, monthly for months 9-12, and 3-monthly for months 13-24).

#### Mentor sessions - structure

During the sessions the mentor will assess progress with each goal since last contact, problem-solve with the family any difficulties arising, and negotiate goals for the next session. Each family will receive a different package of resources over time depending on identified need and there is some scope for resources to be tailored to individual families. Across the period of intervention the mentor may also (in consultation with the expert team) facilitate the introduction of new behavioural goals. The intervention will be conducted in the "spirit" of MI, taking a client-centred collaborative approach, which has been identified as just as important as the specific techniques [21], by adhering to the four general principles of expressing empathy, supporting self efficacy, rolling with resistance and developing discrepancy. MI will be used as required through the life of the intervention, in consultation with the supervisors, when motivation and/or engagement is waning, and when at the transition from one target behaviour to the next (where multiple goals have been identified).

#### Training in MI and assessment

##### Training

Researchers providing the MI completed training in MI over 3 months. Training consisted of a general orientation to the history and development of MI, its use and efficacy through reading key resources, and completion of an online training course in the use of MI in healthcare settings run by the Pacific Centre for Motivation for Change (PCMC, http://www.pacificcmc.com/). Researchers also viewed the BMI^2 ^DVD training system (Resnicow, 2009), which introduces MI and provides specific examples of MI for paediatric obesity in medical settings. Researchers completed a 2-day workshop led by a trainer accredited by the Motivational Interviewing Network of Trainers to further develop skills tailored to providing assessment results using MI for paediatric obesity. Skills were maintained through regular individual and group exercises including role-plays, self-review, and analysis of the content of sessions using the Motivational Interviewing Treatment Integrity (MITI) [70] coding schedule (see below) by the MI supervisors.

##### Supervision

Researchers providing assessment results to participants will attend weekly supervision. Supervisors are clinical psychologists who have completed the training received by the researchers. In addition, supervisors have completed training specific to providing supervision, and completed a day with the MI expert trainer (PCMC), focused on coding MI using the MITI coding system and supervision of MI. The MITI will be used in supervision to provide structured feedback about improving practice, in addition to providing quantitative data about the quality of the MI sessions. Prior to supervision, researchers will review a recording and complete a self-reflection exercise involving an analysis of their adherence to MI, strengths and weaknesses and skills. This exercise has been adapted from MI assessment supervisory tools for enhancing proficiency [71]. During supervision, researchers' self-reflections will be reviewed and specific feedback from the MITI will be provided. Collaboratively, the researcher and supervisor will set specific goals for the following week.

##### Fidelity

The MITI coding system will be used to assess fidelity to MI [70]. The MITI produces a behaviour count of MI-adherent and non-adherent behaviours along with a series of global ratings of dimensions of the interviewers' interactions with the parents that are characteristic of MI (evocation, collaboration, autonomy/support, direction and empathy). All participant sessions will be recorded and coded using the MITI. The MITI will also be applied to a random selection of sessions from the Usual care group to assess the health advisor's style of engagement with parents (global ratings) and the frequency of MI-adherent and non-adherent strategies.

#### Outcome measurements

All measures will be collected at baseline, 12 and 24 months as outlined below. Height, weight, waist circumference and body composition (Tanita BC-418) will be measured as described for screening.

Physical activity will be assessed over seven days using Actigraph accelerometers (GTX3) [72,73]. Parents will be asked to record the time their child got up and went to bed on a daily log. The accelerometers will be worn 24 hours a day (except swimming or bathing) and are attached to belts and placed at the waist in line with the right knee. Measures of sedentary time (television viewing and computer use) will be obtained by questionnaire.

Dietary intake will be assessed using the Children's Dietary Questionnaire [74], which assesses intake patterns over the past week for which intake is recommended (fruit, vegetables, water, reduced fat products) and foods for which intake is discouraged (high fat/sugar foods/non-core foods, sweetened beverages and full fat dairy products). The questionnaire has demonstrated acceptable reliability and relative validity at the group level, for children of this age [74]. Portion size of vegetables, meat and starch-based foods will be assessed by three brief questions which have been validated by duplicate 24-hour recall measures in 7-year old children (Haszard J, unpublished manuscript). Food availability in the home will be assessed using a modified version of the Home Food Inventory [75]. This questionnaire assesses whether a multitude of foods and beverages are present in the home on a specific occasion and creates an obesogenic household food availability score.

The Lifestyle Behaviour Checklist will be used to assess what challenges parents of overweight children face in managing their children's behaviour and how we might address this as part of the tailored package treatment [76]. The Checklist includes 26 weight-related behaviours and asks parents to rate how much of a problem each is, and their confidence in changing each behaviour. Child behaviour will be assessed using the Strengths and Difficulties questionnaire [77].

Quality of life (QoL) will be assessed using the Pediatric Quality of Life Inventory (PedsQL4.0), a validated 23-item questionnaire for children aged 2 to 18 years which assesses physical, emotional, social and school functioning [78]. Decreases in physical and social functioning have been observed in obese compared with normal weight children [79] and are not restricted to the severely obese [80], although community samples often report higher QoL than clinical obese samples [79,81]. Parent-proxy versions of the questionnaire will be used as appropriate (Toddler if 4 years, Young Child if 5-7 years and Child if 8 years) and provide a reliable and valid measure of QoL in children [82]. Furthermore, a recent large representative Australian study [79] demonstrated that children's subjective response to their overweight was very comparable to that of their parents. As utilities have not been determined for PedsQL, quality of life will also be measured using the Health Utilities Index [83,84]. The 40-item version (HUI23P4E.40Q) based on the complementary HUI Mark 2 (seven attributes) and Mark 3 (eight attributes) questionnaires will be answered by a parent/guardian on behalf of each child.

#### Power calculations and statistics

Previous work from our group measured height and weight in more than 1000 children aged 2-18 years and asked parents whether they would be interested in attending a lifestyle programme if one was offered; 45% of parents of overweight children expressed interest (Dawson, unpublished). Thus we wanted to screen enough children to detect a difference in the proportion agreeing to participate in the intervention of 15%, assuming 45% for the Usual care and 60% for the MI feedback condition. It was estimated that 186 children would be required in each group to give 80% power at the 5% level of significance to detect such a difference.

Sample size estimations for the intervention were based on the literature and have been provided for the main outcome variable (BMI z-score [85]) and secondary outcome variables where appropriate (physical activity [86], quality of life [79] and dietary intake [74]). Our study has 90% power at the 5% level of significance to detect differences of i) 0.20 units of BMI z-score with 99 children in each intervention group, ii) 100 counts/minute (14% difference in physical activity) with 35 children in each group and a difference of 20 minutes/day of MVPA with 71 children in each group, and iii) 6 points on the quality of life scale (minimal clinically significant difference is 4.5 points [78]) with 51 children in each group, and iv) a difference in non-core foods diet score of 0.5 with 78 children in each group. Thus a sufficient sample size for all 5 measures would involve approximately 100 children per group. However, allowing for a 20% drop-out increases the numbers required to 125 children per group. Thus all 400 children given feedback will be invited into the intervention to recruit a minimum of 250 (125 per group) into the intervention. A mixed model, adjusting for the baseline measure will be used to analyse the data. The model will test for differences between treatments at the two time periods and the analysis will conform to the CONSORT statement for analysing randomised control trials [87].

## Discussion

The advent of screening for overweight in children prior to school commencement offers a prime opportunity to intervene at a time when only small lifestyle changes may be required to modulate excessive weight gain. However, difficulties surrounding screening, parental response to weight status in children, and general reluctance of health professionals to instigate weight discussions with parents has restricted work in this area. We plan to test whether motivational interviewing offers a suitable technique for working with parents to discuss their child's weight and health status in a manner that is acceptable, and likely to encourage behavioural change, relative to usual care. An important facet of our project is to assess the potential for harm from screening. Recently, the potential for harm has been judged to be low but on the basis of relatively limited evidence [88].

The efficacy of long-term (more than 12 months) weight management in overweight children is also unknown [88]. The second phase of our trial (the intervention) is also consistent with the philosophy of MI and tests whether a patient-centred, mentor-led approach with limited expert support results in effective weight management over a 24-month period, compared with usual care. Cost-effective programmes suitable for incorporation into the health-care environment are urgently needed to reduce excessive weight in young overweight children.

## Competing interests

The authors declare that they have no competing interests.

## Authors' contributions

Principal responsibility for study design and conduct was assumed by RT, AC and DB. RT was responsible for obtaining study funding. All authors contributed to study design and further development of the protocol. AC, DB, AD and LT are responsible for screening and motivational interviewing. SW undertook sample size calculations and designed the statistical analysis. AD, JH and KM were involved in instrument selection, resource development, intervention design and will undertake data collection. BT, JR and ER provide expert input. RT drafted the manuscript and all authors read and commented on drafts and approved the final manuscript.

## Pre-publication history

The pre-publication history for this paper can be accessed here:

http://www.biomedcentral.com/1471-2458/10/271/prepub
